# Supplementary material for: A multimodal Transformer Network for protein-small molecule interactions enhances predictions of kinase inhibition and enzyme-substrate relationships
Source: PLoS Comput Biol. 2024 May 20;20(5):e1012100. doi: 10.1371/journal.pcbi.1012100 (PMC11142704; doi:10.1371/journal.pcbi.1012100)
Supplement: S1 Text — (DOCX) [file pcbi.1012100.s001.docx]

**The ProSmith Transformer Network can be trained with limited computational resources**

The training of Transformer Networks typically demands extensive computational resources. For example, the ESM-1b Transformer Network, designed for protein amino acid sequences, was trained on a computation cluster with 64 individual NVIDIA V100 GPUs, each equipped with 16 GB RAM, over a period of approximately 19 days. In contrast, because of its comparatively small size, we were able to train the ProSmith Transformer Network using a single NVIDIA A100 GPU with 40 GB RAM in a much shorter timeframe; training on the Davis dataset for 100 epochs required only ~22 hours.

In tests on a more affordable GPU -- a single NVIDIA RTX6000 with 24GB RAM -- we obtained the same model performance with an acceptable increase in training time (~39 hours). Thus, interested users can train the ProSmith model for arbitrary protein-small molecule interaction tasks on datasets of up to ~100,000 data points within a reasonable time frame and without the requirement of an extensive GPU infrastructure. Such future applications are greatly facilitated by the user-friendly Python functions provided on our GitHub repository.
