# Supplementary material for: A multimodal Transformer Network for protein-small molecule interactions enhances predictions of kinase inhibition and enzyme-substrate relationships
Source: PLoS Comput Biol. 2024 May 20;20(5):e1012100. doi: 10.1371/journal.pcbi.1012100 (PMC11142704; doi:10.1371/journal.pcbi.1012100)
Supplement: Attachment — Submitted filename: Response2Reviewers.pdf [file pcbi.1012100.s012.pdf]

Reviewer #1:

This manuscript proposed a new deep learning model for protein-small molecule interaction prediction. The authors argued that previous methods, which usually combined the representations of proteins and small molecules in the embedding space, might not be able to capture the fine-grained interaction patterns between amino acids and molecular atoms. To address this, they proposed a Transformer-based method that combines the information in the input space, i.e., concatenating the amino acid sequence and SMILES string. The model then relies on the self-attention layer of Transformer to capture the interactions between amino acids and molecular atoms. The proposed method was evaluated on three tasks, including drug-target binding affinity prediction, enzyme-substrate interaction prediction, and enzyme-substrate affinities prediction, with performance improvements over existing methods.

Overall, the manuscript proposed a deep learning model with good results on three different evaluation tasks. I have some major comments about the model design and evaluation, which would help strengthen the work:

Response: We thank the reviewer for the positive assessment and the detailed and thoughtful comments provided. We greatly appreciate the time and effort the reviewer took to provide this feedback, and we found the comments to be extremely helpful, especially in identifying areas that require further analysis and comparison. We believe that the feedback has helped us to significantly improve our manuscript.

Major comments:

1. Comparison to per-protein or per-molecule models. A closely related work (Ref 25 in the manuscript) found that training an individual specific model for each protein/molecule had better performance than training a one-for-all model that used a protein-molecule pair as input for all data. Ref 25 also found that a simple method that fits a ridge regression model on ESM embeddings or molecular fingerprints outperformed deep learning-based models. Can the authors compare the proposed model to the per-protein/-molecule models based on ridge regression in Ref 25?

Response: Our goal was to develop a model that could generalize to previously unseen proteins and protein families. In contrast, Goldmann et al. (ref. 25) investigated prediction models for well-studied enzyme families with large amounts of available experimental data. While we have shown in a previous study that our general approach can also outperform models designed specifically for individual enzyme families (https://doi.org/10.1038/s41467-023-38347-2), we agree that if one is interested in a small number of protein families with sufficient experimental data, it is conceivable that family-specific models such as those described in Ref. 25 could lead to superior results.

Action: We discuss in the revised manuscript that family-specific models may lead to superior results when large amounts of experimental data are available (ll. 424ff.):

> A previous study showed that training a specific model for each protein or each small molecule can lead to superior performance compared to a more general model trained for handling different proteins and small molecules [25]. However, such specific models can only be trained for proteins and small molecules for which large amounts of experimental data are available. In contrast, our goal was to develop a model that can generalize to previously unseen proteins, protein families, and small molecules. In the vast majority of test cases in our study, a protein- or small molecule-specific model as described by Goldman et al. [25] cannot be fitted due to a lack of training data. While we have shown in a previous study that a general approach can outperform state-of-the-art models designed specifically for individual enzyme families [18], if one is interested in specific protein families or small molecules with sufficient experimental data, it is conceivable that specific models could lead to superior results.

2. Investigation of model design. The proposed method used a two-stage approach for the prediction: i) first train a Transformer on the training set, then ii) extract the Transformer's hidden embedding (the 'cls' embedding), combined with ESM and ChemBERTa2 embeddings, to train another gradient boosting model for the final prediction. Could the authors further elaborate/discuss why a Transformer + boosting tree approach was used, instead of an end-on-end approach using the Transformer? The authors cited two papers that showed Transformer + boosting tree had better prediction performance, but I would like to see a more specific discussion on why and

in which scenarios (e.g., data size, modality, etc) the Transformer + boosting tree approach would be better than an end-to-end Transformer model? My take from the manuscript is that the Transformer is a good feature extractor, and the gradient boosting tree is a good predictor, but the performance difference between the two-stage and the end-to-end versions of the proposed model is unclear. The authors are suggested to compare with an end-to-end version of their model, e.g., replacing the boosting tree with a multi-layer perceptron (MLP) on top of the embeddings from Transformer, ESM, and ChemBERTa2, and training it end-to-end. It would also be interesting to investigate if varying the number of layers or dimensions of the MLP would lead to a performance close to the gradient boosting tree.

Response: We agree that we haven't actually shown that using gradient boosting models on top of the joint embeddings is better than the end-to-end Transformer Network with a multi-layer perceptron (MLP) on top of the learned embeddings.

Action: In the revised manuscript, we evaluate the end-to-end trained Transformer Network with an MLP on top of the learned joint protein-small molecule representation for the enzyme-substrate prediction task. We compare this performance to the gradient boosting model that only uses the learned joint protein-small molecule embeddings as its input. To facilitate a fair comparison, we vary the number of layers and dimensions of the multi-layer perceptron as an additional hyperparameter (ll. 375ff.):

> For the ProSmith model, we first train a multimodal Transformer Network end-to-end: input is the protein amino acid sequence and a string representing the small molecule structure, and output is the target feature — e.g., whether protein and small molecule form an enzyme-substrate pair. The final ProSmith model does not use these predictions directly, but instead uses the learned joint protein-small molecule representations to train gradient boosting models. This strategy was motivated by previous studies that showed superior results when adding a gradient boosting step [18, 37]. To investigate whether this additional step indeed contributed to the superior performance of ProSmith, we re-examined the enzyme-substrate prediction task, comparing the model performance of directly using the end-to-end trained multimodal Transformer Network with that of a gradient boosting model that takes the learned joint protein-small molecule representation from this Network as input. The end-to-end trained Transformer network uses a fully connected neural network to process the learned protein-small molecule representations. To facilitate a fair comparison, we explored different numbers of hidden layers and dimension sizes, testing all combinations of one or two hidden layers with hidden dimension sizes of 32, 64, or 128 (Supplementary Table 7). In comparison to the full ProSmith model, the best-performing Transformer Network architecture (2 layers with 128 nodes) decreased accuracy from 93.3% to 92.2%, MCC from 0.84 to 0.81, and ROC-AUC from 0.963 to 0.946. Thus, using a gradient boosting model on top of the learned representation indeed improves model performance.

3. Ablation study. The authors claimed that concatenating features in the input space is better than concatenating in the embedding space. Although some of the compared baseline methods (e.g., GraphDTA) used the latter approach, those methods did not use the same capable Transformer model. As a more direct test, could the authors compare a variant of their proposed method that combines features in the embedding space, e.g., using two separate Transformers to process the ESM and ChemBERTa embeddings, concatenating them, and then pass them to the predictor?

Response: Again, we agree with the reviewer that we should show that the advantage of our model is actually the multimodal model and not predominantly the task-specific embeddings.

Action: As suggested by the reviewer, we now compare our model to a variant that uses two separate, parallel Transformers to process the ESM and ChemBERTa embeddings, and concatenating them only after they passed through the Transformer layers. We then used this concatenate vectors as input for the gradient boosting models and compared performance to the full ProSmith model (ll. 392ff.):

> We argue that the main architectural advancement of ProSmith is its ability to process small molecule and protein information in the same input string, which facilitates the exchange of information between protein and small molecule while generating a joint numerical representation. To test whether this is indeed the case and whether the improved performance is not simply due to training a Transformer Network end-to-end, we trained an alternative model for the enzyme-substrate prediction task that uses two separate Transformers to process the protein amino acid sequence and the small molecule SMILES string separately but in parallel. We concatenated the protein and small molecule representations only after they passed through the Transformer Networks, and used the resulting vector as input to a fully connected neural network. As for the ProSmith model, this model was first trained end-to-end before extracting the protein and small molecule representations and using them as input for gradient boosting models. This process is identical to our ProSmith model, except that the model does not allow information exchange between protein and small molecule while generating the numerical representations. Compared to the full ProSmith model, this alternative model decreased accuracy from 94.2% to 93.4%, ROC-AUC from 0.972 to 0.966, and MCC from 0.848 to 0.834. These results indicate that a major architectural advancement of the ProSmith model is indeed its ability to simultaneously process protein and small molecule information in the same input sequence using the multimodal transformer network.

4. Table 3 shows that ProSmith outperformed ESP, but should we attribute the improvement to the ML model or the data augmentation technique? Please clarify with an ablation study, e.g., adding another line in the table of ProSmith's result without data augmentation.

Response: We used the exact same datasets for training and evaluation in the current manuscript and in the ESP paper. Hence, the improvement is not due to a different data augmentation strategy, and we can indeed directly compare both model performances.

Action: We make this point more explicit in the revised manuscript (ll. 282f.):

> To train and test ProSmith for the same enzyme-substrate prediction task, we use training, validation, and test datasets that are identical to the datasets used in the ESP study.

Minor comments:

5. Abstract: "The resulting predictions outperform all previous models" -- I suggest changing "all" to another word since not all existing methods were compared in this work.

Action: We substituted "all previous" with "recently published state-of-the-art":

> The resulting predictions outperform recently published state-of-the-art models for predicting protein-small molecule interactions across three diverse tasks: predicting kinase inhibitions; inferring potential substrates for enzymes; and predicting Michaelis constants KM.

6. Fig 2: can we also add the leading baselines' results in this figure? It would better illustrate the performance gap between the proposed method and baselines in the low-data regime.

Response: We agree that it would indeed be desirable to show comparisons to previous results in Figure 2. Unfortunately, predictions for all test data points of the leading baseline are not available, only final performance metrics are published. Therefore, we cannot add a comparison to the leading baseline in Figure 2.

Action: None

7. Line 244: the authors claimed that their previous ESP model is “the only general model for the prediction of enzyme-substrate pairs”. Could the authors clarify what is a “general model” and why their model is the only general model? How about the models developed in the following PLOS CB paper (cited as Ref 25 in the manuscript): Goldman et al. (2022). Machine learning modeling of family wide enzyme-substrate specificity screens. PLoS computational biology.

Response: By "general enzyme-substrate prediction model", we refer to a model that is, in principle, capable of predicting potential substrates for any arbitrary enzyme. In contrast, the models in Reference 25 are fitted to specific enzyme families and therefore cannot be applied to enzymes outside of these families.

Action: In the revised manuscript, we now define what we mean by "general prediction model" (ll. 278f.):

> By ”general model” we refer to a model that, in principle, can be applied to any enzyme without further adaptation.

8. For Figures 2 & 3, can you also show the figures as a function of drug similarity & number of training samples with identical proteins?

Response: For the prediction of drug-target affinity predictions this is indeed possible. For the enzyme-substrate pair prediction, training and test sets are constructed such that no protein is present in both sets – there are no identical proteins. Hence, we cannot calculate how model performance changes based on the number of times a protein is present in the training set. However, we can estimate how model performance depends on substrate similarity compared to the substrates used for training.

Action: For the drug-target affinity prediction task, we now calculate, in addition to Figure 2, model performance for drugs based on a similarity score compared to training drugs, and we calculated model performance based on the number of times a protein occurs in the training set. For the enzyme-substrate prediction task, for all test substrates that were not present in the training set, we compute how model performance depends on the maximal substrate similarity score compared to the training substrates. We plot the results in Supplementary Figures (ll. 259ff. & ll. 307ff.):

> In addition, we evaluated model performance for test drugs that were not present in the training set based on their maximum similarity compared to all training drugs (Supplementary Figure 1a). We found that model performance for drugs increases when similar drugs are present in the training set: While model performance is low for drugs with a maximum similarity score below 0.6 compared to any training drug, good prediction results can be achieved for unseen drugs with similarity scores above 0.6. If a test protein was already present in the training set, we also analyzed

how model performance changes based on the number of occurrences of the protein in the training set (Supplementary Figure 1b). However, we did not find a strong correlation between protein occurrence in the training set and model performance. This may not be surprising, since model performance is already high when at least one highly similar protein is present in the training set (Figure 2b).

&

For test substrates that were not present in the training set, we further investigated how model performance depends on their maximum similarity compared to all training substrates (Supplementary Figure 2). As in the drug target prediction task, we find that model performance increases when structurally similar substrates are present in the training set: Model performance is low for unseen substrates with a maximum similarity score below 0.6 compared to all training substrates, but moderate prediction results are obtained for substrates with similarity scores above 0.6.

9. (a) For enzyme-substrate pair/affinity tasks, were the data splits based on random split? How about those cold-start splits used in the drug-target task?

Response: For the enzyme-substrate prediction task, the data set was split randomly while ensuring a maximum sequence identity of 80% between training and test enzymes. This is a cold split for the proteins, since the entire test set contains only unseen enzymes. For the substrates, an estimate of the performance on a cold split can be seen in Figure 3a in the left bucket, which contains only test data points where the metabolite did not occur in the training set.

For the K_M prediction task, the data set was randomly split into 80% training data and 20% test data, ensuring that the same enzyme-substrate pair would not be in the training and test sets. An estimate for the cold split scenarios is shown in Supplementary Figure 1; for the substrates in Supplementary Figure 1a, the subset where the substrate was not present in the training set, and for the enzymes in Supplementary Figure 1b, all enzymes with sequence identity below 100%.

Action: For both datasets, we added sentences describing the splitting procedures as performed in the original publications (ll. 285ff. & ll. 328):

The dataset was divided into 80% training data and 20% test data, ensuring that no enzyme in the test set has a sequence identity greater than 80% compared to any enzyme in the training set.

&

> We adopted the same split used in that study, which divided the KM dataset into 80% training data and 20% test data, while ensuring that the same enzyme-substrate pair would not be in the training and test sets.

10. Table 4: please explain why the MSE value for ENKIE (2022) was missing.

Response: Unfortunately, the ENKIE study did not publish the MSE metric; the authors also did not provide the predictions for the K_M test dataset, so that we cannot calculate the MSE ourselves.

Action: In the revised manuscript, we now state why the MSE is missing for the ENKIE study (ll. 340ff.):

> We cannot compare the MSE between ProSmith and the ENKIE model, as Ref. [13] does not report this metric or the individual predictions for the KM test set.

Reviewer #2:

To simultaneously process protein amino acid sequences and small molecule strings in the same input, the proposed method map both types of information to the same embedding space, creating input for a transformer network and gradient boosting models. The methods is used for a number of important applications, demonstrating the generality of transformer networks and achieving results that are competitive or better than the ones produced by earlier and more specific methods. These are valuable results.

Response: We thank the reviewer for this positive assessment and helpful feedback. The reviewer's comments helped us to clarify the aim and scope of this study in the revised manuscript.

1. The paper has clear potential value, assuming the method will be used in the future. Nevertheless, it is disappointing that the entire research is devoid of any biology. To see some interesting applications would substantially increase the value of the contributions.

Response: Our manuscript was submitted for the ‘Methods’ section of PLOS Comp Biol. The aim of our work is simply to develop, describe, and validate a new method to predict protein-small molecule interactions. While we agree that additional biological insights could

make the manuscript more interesting to a broader audience, such insights are beyond the scope of our work.

Action: None

> 2. What is particularly disappointing is that the authors refer to the Davis data set (ref 44) as “the dataset comprises 30 056 data points, consisting of binding affinities for pairs of 72 drugs (small molecules) and 442 target proteins, measured as dissociation constants Kd (in units of nM)”. The title of ref 44 is “Comprehensive analysis of kinase inhibitor selectivity”, and the dataset is entirely specific to kinase inhibitors. This has two implications. First, the results are most likely more relevant to these type of interactions rather than generally to protein-drug interactions as suggested by Kroll et al. Second, the results might have provided some interesting insights on the properties of kinase inhibition. However, the word “kinase” is not even mentioned in the Kroll paper. So it seems the authors specifically wants to move away from any implication concerning biology, possibly to imply more generality than provided by the analysis of a kinase-inhibitor dataset. Why not at least mention the specific biology problem, i.e., kinase inhibitor specificity?

Response: We agree that it is important to emphasize that the Davis dataset describes kinase inhibition rather than general drug-target interactions, as noted by the reviewer. However, most druggable proteins are kinases (https://doi.org/10.3389/fcell.2022.942500). Therefore, kinase datasets are the most commonly used data for evaluating target inhibition predictions – in particular the Davis dataset (see for example https://doi.org/10.1093/bioinformatics/btad355, https://doi.org/10.1093/bioinformatics/btaa921, https://doi.org/10.1093/bib/bbab506). Accordingly, we consider the Davis dataset to be an appropriate representative of drug-target affinity data also for our study.

While we agree that an in-depth analysis of our predictions might lead to some insights into the properties of kinase inhibition, such analyses were beyond the scope of our paper.

Action: When referring to the Davis dataset, we now emphasize that our analysis contains information about kinase inhibition rather than drug-target affinities in general. We have changed the title from "A multimodal Transformer Network for protein-small molecule interactions enhances drug-target affinity and enzyme-substrate predictions" to "A multimodal Transformer Network for protein-small molecule interactions enhances predictions of kinase inhibition and enzyme-substrate relationships", and we have adjusted the description of the dataset and the prediction task in the Abstract, Introduction, and corresponding Results sections (l. 10, ll. 71ff.. & ll. 178ff.):

> The resulting predictions outperform recently published state-of-the-art models for predicting protein-small molecule interactions across three diverse tasks: predicting kinase inhibitions; inferring potential substrates for enzymes; and predicting Michaelis constants KM.

&

We show that ProSmith outperforms previous models in predicting protein-small molecule interactions across three diverse tasks: predicting the affinity between protein kinases and drugs [1–12]; evaluating whether a small molecule is a natural substrate for a given enzyme [18–26]; and predicting the Michaelis constant KM of an enzyme for its substrate [13–17].

&

Here, we assess the performance of ProSmith on one of the most widely used datasets for validating drug-target affinity prediction models, the Davis dataset [44]. The Davis dataset comprises 30 056 data points, consisting of binding affinities for pairs of 72 drugs (small molecules) and 442 target protein kinases, measured as dissociation constants Kd (in units of nM). To create target values for ProSmith, we follow previous prediction methods by using log-transformed values, defined as pKd = − log10(Kd / 10^9) nM. The resulting values range from 5.0 to 10.8. Since the Davis dataset consists only of protein kinases, its analysis does not allow direct conclusions about ProSmith's ability to predict drug affinities for other protein families. However, most druggable proteins are kinases [45], and therefore kinase datasets are the most commonly used data for evaluating target inhibition predictions.

3.

(a) While I recognize the contributions of the work for general computer science for demonstrating the application of the same neural network to a variety of problems (which is not entirely novel),

(b) I have some doubts whether in its present form this paper makes contributions to computational biology. More generally, it really needs discussing some applications with biological value.

Response (a): Our aim was not to demonstrate that the same neural network can be applied to distinct problems. Instead, we aim to introduce and validate the model architecture of ProSmith, which: (i) is novel, as comparable multimodal neural networks have not been applied for molecular interaction prediction tasks before; and (ii) can indeed be applied across a variety of problems that involve interactions between proteins and small molecules.

Response (b): We agree with the reviewer that our manuscript does not contribute to our understanding of biological systems. Instead, it contributes a novel and powerful method to make biologically relevant predictions. Thus, we provide methodological rather than biological insights. According to the PLOS Comp Biol "Journal Information" page, "Research articles specifically designated as Methods papers should describe outstanding methods of exceptional importance that have been shown, or have the promise to provide new biological insights." We are convinced of that promise, as also suggested by the reviewer in the

discussion of the Davis dataset (“the results might have provided some interesting insights on the properties of kinase inhibition”). We are thus convinced that our manuscript is within the scope of PLOS Comp Biol’s Methods section, but ultimately that is an editorial decision.

Action: In the introduction, we now emphasize the aims of our study more clearly. We make explicit that our study’s aim is to provide a method for gaining biological insights rather than biological insights *per se* (ll. 68ff.)

> This study presents the first multimodal Transformer Network capable of processing molecules with different modalities. The goal of this study is not to provide new biological insights into molecular interactions per se, but rather to demonstrate that the developed method can be successfully applied to a variety of prediction problems involving interactions between proteins and small molecules.
